# Buffalo nasal odorant-binding protein (bunOBP) and its structural evaluation with putative pheromones

**DOI:** 10.1038/s41598-018-27550-7

**Published:** 2018-06-19

**Authors:** Subramanian Muthukumar, Durairaj Rajesh, Ramu Muthu Selvam, Ganesan Saibaba, Suvaiyarasan Suvaithenamudhan, Mohammad Abdulkader Akbarsha, Parasuraman Padmanabhan, Balazs Gulyas, Govindaraju Archunan

**Affiliations:** 1 Center for Pheromone Technology (CPT), Department of Animal Science, Bharathidasan University, Tiruchirappalli, 620024 Tamil Nadu India; 2 Center for Animal Research, Training and Services (CAReTS), Central Inter-Disciplinary Research Facility (CIDRF), Mahatma Gandhi Medical College & Research Institute campus, Pillaiyarkuppam, Puducherry 607402 India; 3 Research Institute in Semiochemistry and Applied Ethology (IRSEA), Quartier Salignan, 84400 APT France; 4 Present Address: Winro Research Institute of Biological Sciences, winro Science Research Foundation, Tiruchirappalli, 620 007 Tamil Nadu India; 5 Department of Bioinformatics, Bharathidasan University, Tiruchirappalli, 620024 Tamil Nadu India; 6 Mahatma Gandhi-Doerenkamp Centre, and Department of Animal Science, Bharathidasan University, Tiruchirappalli, 620024 Tamil Nadu India; 7 Present Address: National College (Autonomous), Tiruchirappalli, 620001 Tamil Nadu India; 8 Lee Kong Chian School of Medicine, Nanyang Technological University, Singapore, 636921 Singapore

## Abstract

Pheromones are odoriferous volatile chemical cues produced by animals for communication among conspecifics so as to regulate their social behaviors. In general, the odor compounds are recognized by receptors in the nasal cavity. Odorant-binding protein (OBP), a lipocalin family protein, mediates the air-borne odor cues to nasal receptors through nasal mucus. The presence of OBP in several mammalian species is well documented but to-date there is no report of a nasal OBP in buffalo. Hence, the present study was undertaken to investigate if OBP is present in buffalo nasal mucus. Uni- and two-dimensional gel electrophoresis of the nasal mucus suggested the presence of OBP, which was confirmed using mass spectrometry. *In silico* homology model of the OBP was generated and its structural similarity with other mammalian OBPs was assessed. Finally, molecular-docking and -dynamics simulations analysis revealed the efficiency of buffalo nasal OBP (bunOBP) to bind with buffalo pheromones as well as other reported chemical cues. Taken together, the occurrence of nasal OBP in buffalo and its putative role in odor binding are reported for the first time. The potential association of this protein with estrus-specific volatiles could be taken to advantage for non-invasive detection of estrus in buffaloes.

## Introduction

Odorant binding proteins (OBP), a subclass of soluble proteins called outlier lipocalins, has been reported as a major shuttle for odor perception, olfactory stimulus and chemical communication, especially in insects and mammals. Many isoforms of OBP are known which have been isolated, purified and characterized from the nasal mucus of several mammals such as bovine, porcine, rabbit, rodents, etc.^1–6^. Basically, these are 19–23 kDa acidic proteins, which are produced in sero-mucus glands of the respiratory and olfactory epithelia. The concentration of OBP is about 1% of the total soluble proteins present in the mucus of the nasal mucosa^1,3^. Generally, OBP in the nasal region is concerned with receiving, processing and presenting odorant molecules to the specific target(s), which is an odorant receptor. As members of the lipocalin superfamily OBPs have many conserved residues and similar tertiary structural features viz., N-terminal 3_10_ helix and single eight-stranded continuously hydrogen-bonded antiparallel β-barrel, which encloses an internal ligand-binding site for enhancing the ability to bind the ligands of different sizes, shapes and chemical properties^7^.

Certain OBPs are specifically involved in aspects of pheromone/odor communication in mammalian species through body fluids^8^. Such protein are MUP (Major Urinary Proteins) in mouse^9^, urinary and/or preputial gland α2u globulin in rat^10,11^, aphrodisin in hamster vaginal secretion^12^, salivary lipocalin in boar^13^, OBP in buffalo^14^ and panda^15^. The pheromone binding-OBP in the body fluids and the odor binding-OBP in nasal mucus are identical in structure although they may differ in chemical and physiological properties^7^.

The nasal OBP has been implicated in major roles viz., (i) trapping odorants and presenting them to the olfactory receptor (OR) through the mucus barrier^16,17^, (ii) removal of odor molecule from OR after the signal transduction is over as well as when the concentration of the odor molecule is higher than required to elicit the response so as to avoid saturation of the OR; and (iii) protection of nasal mucosa from oxidative stress^18,19^. OBP of mammals was first discovered in nasal mucus and epithelium of cow where it is present in very low concentrations^1,20^. Subsequently, studies have expounded the functional significance of OBP in the bovine, which in computational and crystal analyses revealed a structure that is favorable for odor binding. The first 3D structure analysis of bOBP, reported at 2.4 Å, showed that the bovine OBP molecule is a dimer in which the C-terminal domain occurring at residues 125–159 swaps between the two monomers, a feature very specific to bovine species^21,22^. A similar structure has been previously reported, adopting X-ray crystallography, for human retinol-binding protein^23^ and rodent urinary protein^10^.

Thus, even though there are many reports pertaining to bovine OBP, highlighting the structure and ligand-binding efficiency, there is to-date no information on a buffalo nasal OBP (bunOBP). Understanding pheromone communication and role of OBP in it will be of interest in the light of the fact that in the buffalo there is no perceptible outward symptoms of estrus. In other words, buffalo is a silent estrus/heat animal. Therefore, the farmers have difficulty in detecting the estrus phase in buffalo so as to bring up coitus and/or artificial insemination unlike cow, the estrus symptoms of which are better expressed. However, during estrus, the she buffalo excretes nano-quantities of sex pheromones, which attract the buffalo bull and facilitate mating. A set of estrus-specific pheromone compounds, p-cresol and oleic acid, have been reported in buffalo feces and urine^24–26^. Thus, it is comprehensible that buffalo bull is able to detect the estrus phase of she-buffalo through specific pheromone cues with a role to his nasal OBP, for which there is no direct evidence until now. Therefore, in the present work we undertook a gel-based proteomics study and report the presence of OBP in the nasal mucus of buffalo. Additionally, the comparative homology model for bunOBP has been developed by computational methods. Further, the binding efficiency of this bunOBP with the various possible chemical cues, including from buffalo, was assessed adopting molecular-docking and -dynamics simulations to infer the specificity of protein-ligand interaction in buffalo sexual communication in the present context.

## Results

### 1D (Single dimensional) Gel Electrophoresis

**Figure 1:**
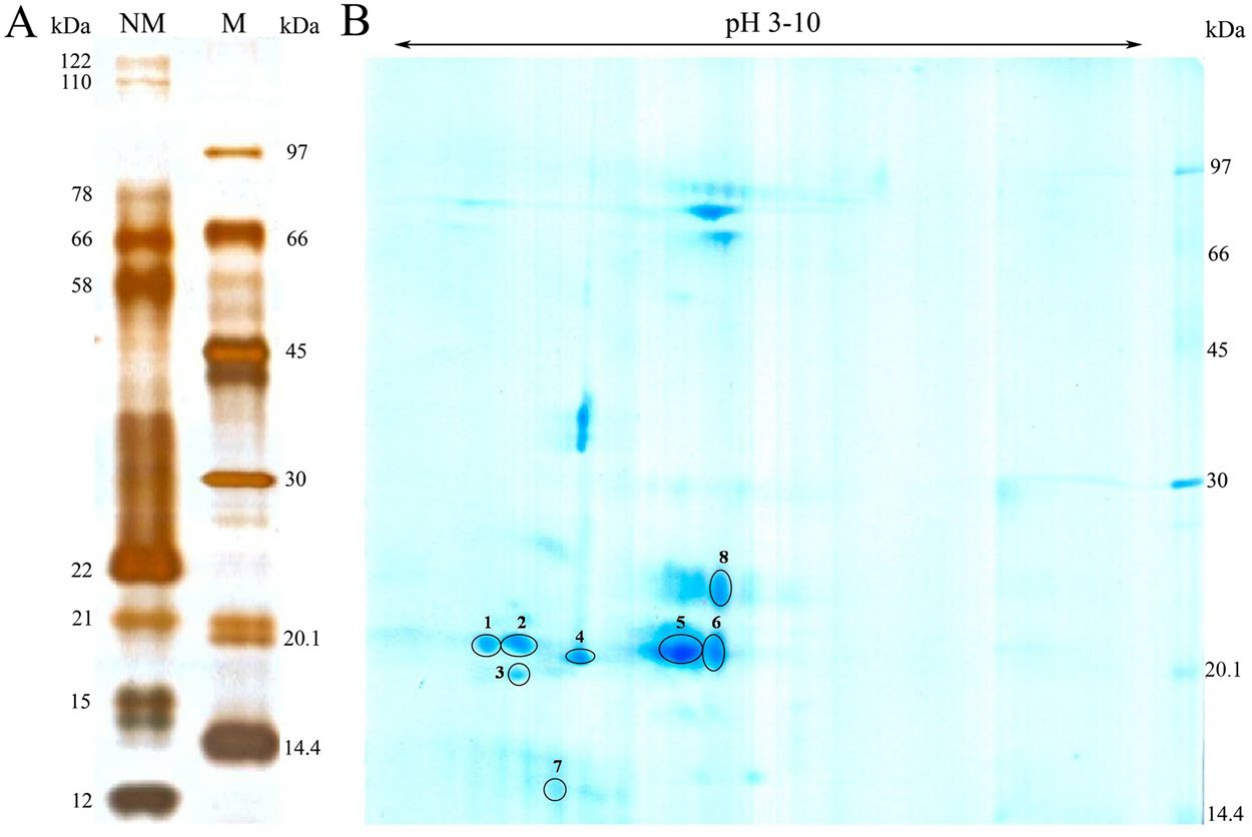
Gel electrophoresis. (**A**) 1D protein profile of buffalo nasal mucus. 1 μL of molecular weight marker proteins (M), 5 μg of nasal mucus protein (NM) were separated by 12.5% SDS-PAGE. (**B)** 2D protein profile of buffalo nasal mucus. Nasal mucus proteins (50 μg) were separated in the first dimension (x-axis) for which the pI range was 3–10, and in the second dimension (y-axis) by SDS-PAGE for molecular weight and then visualized by CBB G250 staining.

The nasal mucus proteins were separated in 12.5% resolving gel. The molecular weights (MW) of the proteins that resolved ranged from 12 to122 kDa in glutaraldehyde-silver stained gel. The 12 to 22 kDa low molecular weight proteins were highly prominent when compared to the high molecular weight proteins (Fig. 1A).

### 2D (Two dimensional) Gel Electrophoresis

The 2D separation of buffalo nasal mucus protein revealed protein spots ranging in MW from 14 to 96 kDa. Coomassie-stained 11 cm strip, at pI 3–10 (broad range), revealed at least fifteen protein spots of which the low molecular weight proteins in the range 14 to 22 kDa were highly prominent (Fig. 1B).

### Mass Spectrometry Analysis

**Figure 2:**
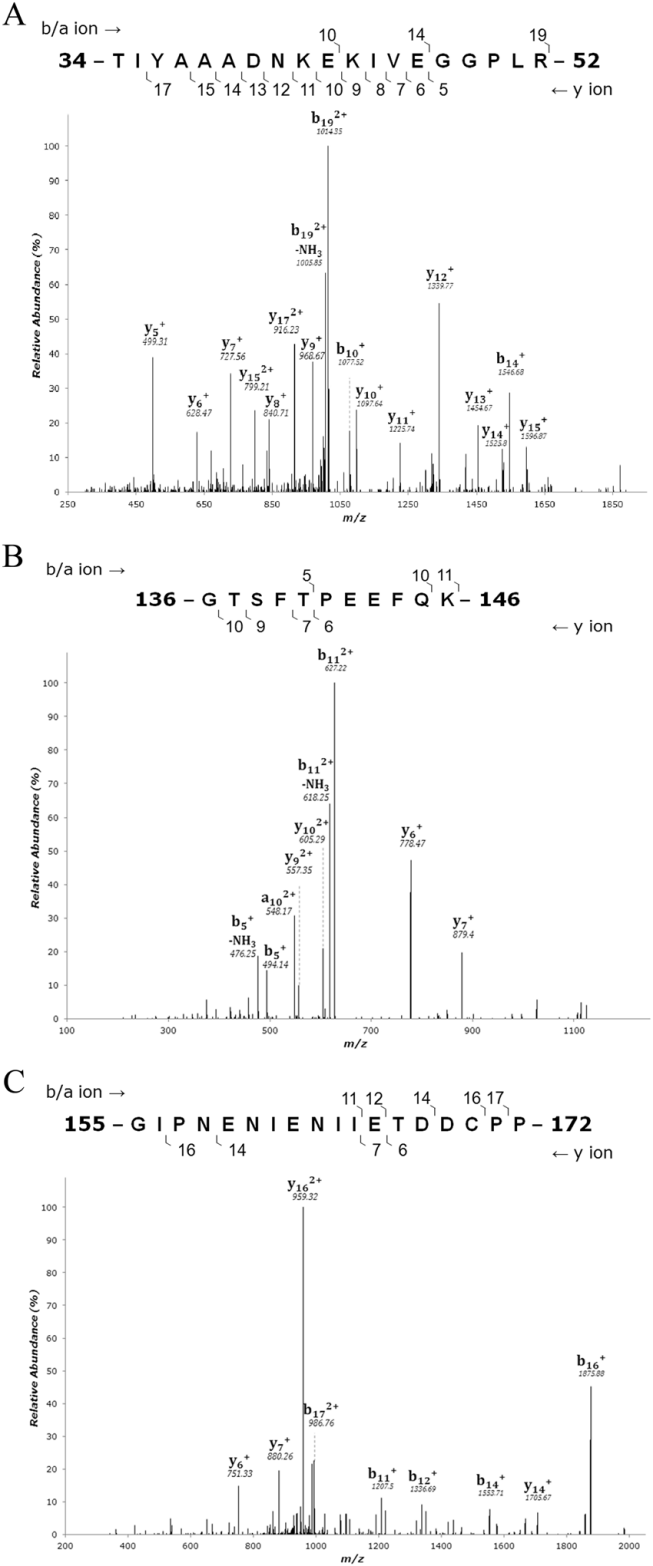
Tandem mass spectrum of nasal mucus OBP (Spot 4). The figure shows the three representative MS/MS peptide sequences of buffalo nasal mucus OBP. The peptides are (**A**) 34-TIYAAADNKEKIVEGGPLR-52 (**B**) 136-GTSFTPEEFQK-146 (**C**) 155-GIPNENIENIIETDDCPP-172.

**Figure 3:**
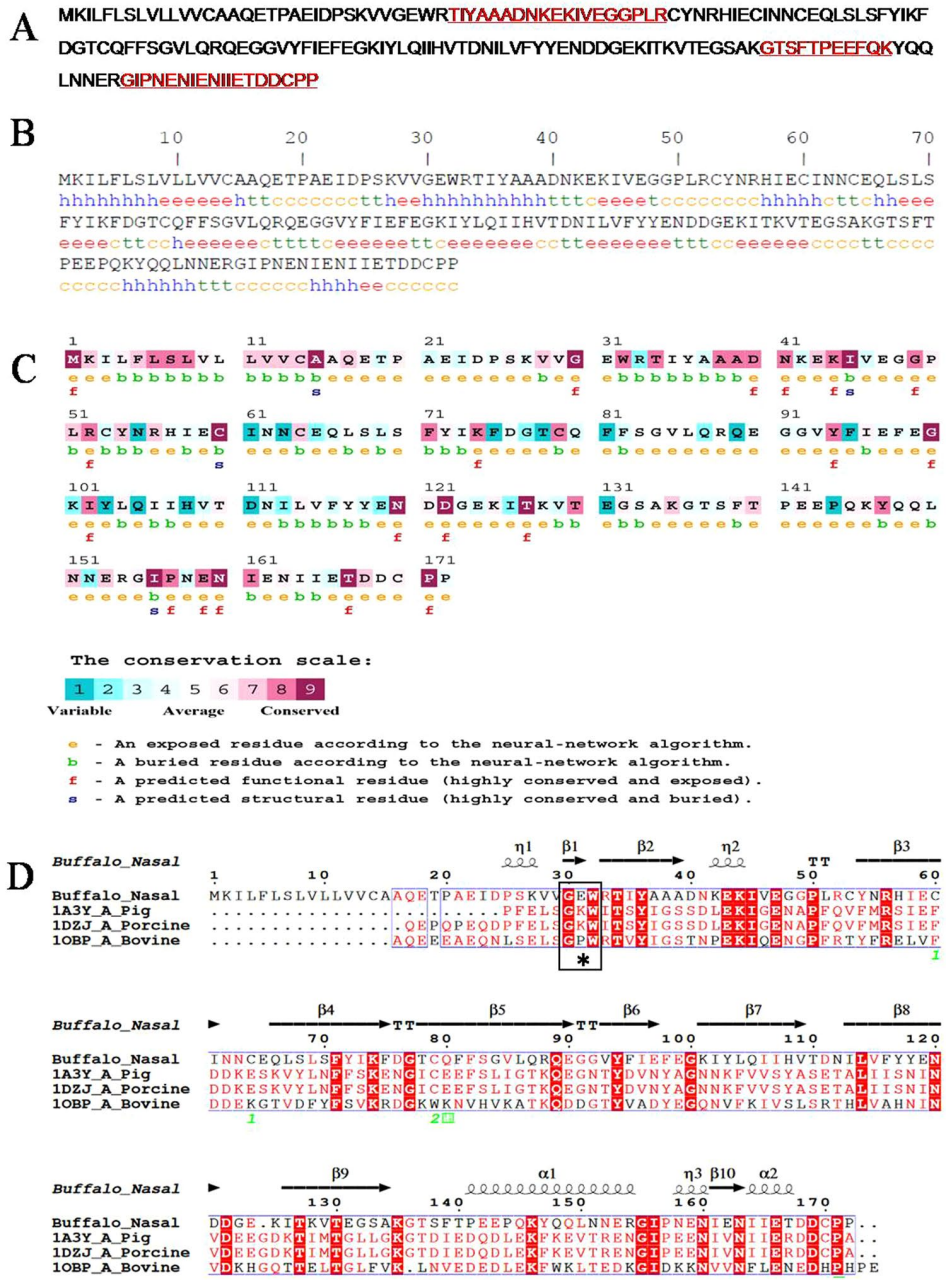
Sequence analysis of bunOBP. (**A**) Matched representative peptides of bunOBP are highlighted in underlined bold red. (**B**) Predicted Secondary structure of bunOBP. The total sequence length is 172 residues. The sequence is characterized by the following (i) extended strand (Ee), (ii) random coil (Cc), (iii) alpha helix (Hh), and (iv) beta turn (Tt). (**C**) The map shows the conserved amino acids in bunOBP with other mammalian OBPs with a conservation scale. (**D**) Multiple sequence alignment. The bunOBP sequence is matched with nasal OBP of other mammals showing several matched peptides. The most reported -GXW-* motif region is present in the bunOBP.

In order to identify the OBP, Coomassie brilliant blue (CBB) stained 2D gel spots at 14 to 22 kDa were subjected to mass spectrometry analysis. The spot numbers 2, 3 and 4 (MW 21 kDa) of buffalo nasal mucus protein were matched with the bovine OBP. Among them the spot 4 at 21 kDa showed up to be the OBP. Three peptides matched bovine OBP and the same *de novo* sequences were plotted (Fig. 2A–C), and the buffalo nasal OBP protein sequence was employed to homology modeling (Fig. 3A).

### Properties of buffalo nasal OBP

ProtParam tool was used to analyze the amino acid (AA) sequence and physico-chemical properties of bunOBP (buffalo nasal OBP) via Expasy bioinformatics resource portal. It revealed 172 residues. The lowest molecular weight was 19604.1 Da and the theoretical pI was 4.57. There were 15 positively charged residues (Arg and Lys combined) and 27 negatively charged residues (Asp and Glu combined) in the sequence. The aliphatic index was calculated as 88.37. The grand average of hydropathicity (GRAVY) was calculated as −0.226. Glutamine (Glu_E) was present in highest number, up to 19, with the percentage of residue being 11.0%, and Histidine (His_H) was present at 1.20%. However, pyrrolysine and selenocysteine were totally absent (0.00%) (Table S1).

### Secondary Structure Prediction

The random coil (Cc) was found to be the most frequent (36.05%), followed by extended strand (Ee) (33.14%) and alpha helix (Hh) (20.35%). The beta turn (Tt) was far too limited (10.47%) in the protein sequence. On the other hand Pi helix, beta bridge and bend region were absent (Fig. 3B).

### Residue Conservation Map

ConSurf score scale ranges from 1 to 9, where score 1 depicts variable as light blue color, score 5 depicts average as white color and score 9 depicts conserved as dark/light pink color. In the present study, the results revealed that in the bunOBP sequence most of the amino acids are highly conserved, and the functional regions are with score 9 in the conservation scale (Fig. 3C, Fig. S1).

### MSA (Multiple Sequence Alignment)

The sequence alignments revealed a number of identical residues with significant conserved motif regions. The bunOBP sequence had 70.51% matches with bovine lipocalin (PDB id: 1BJ7_A) and 40% matches with bovine nasal mucosal OBP (PDB id: 1OBP). The -GXW-*motif region, a signature of lipocalin superfamily, is functionally conserved and acts as an evolutionarily significant amino acid to authenticate the protein diversity^3^. The visualization and representation of MSA was made using ESPript server with bovine, pig and porcine OBP sequences (Fig. 3D).

### Phylogenetic Analysis

**Figure 4:**
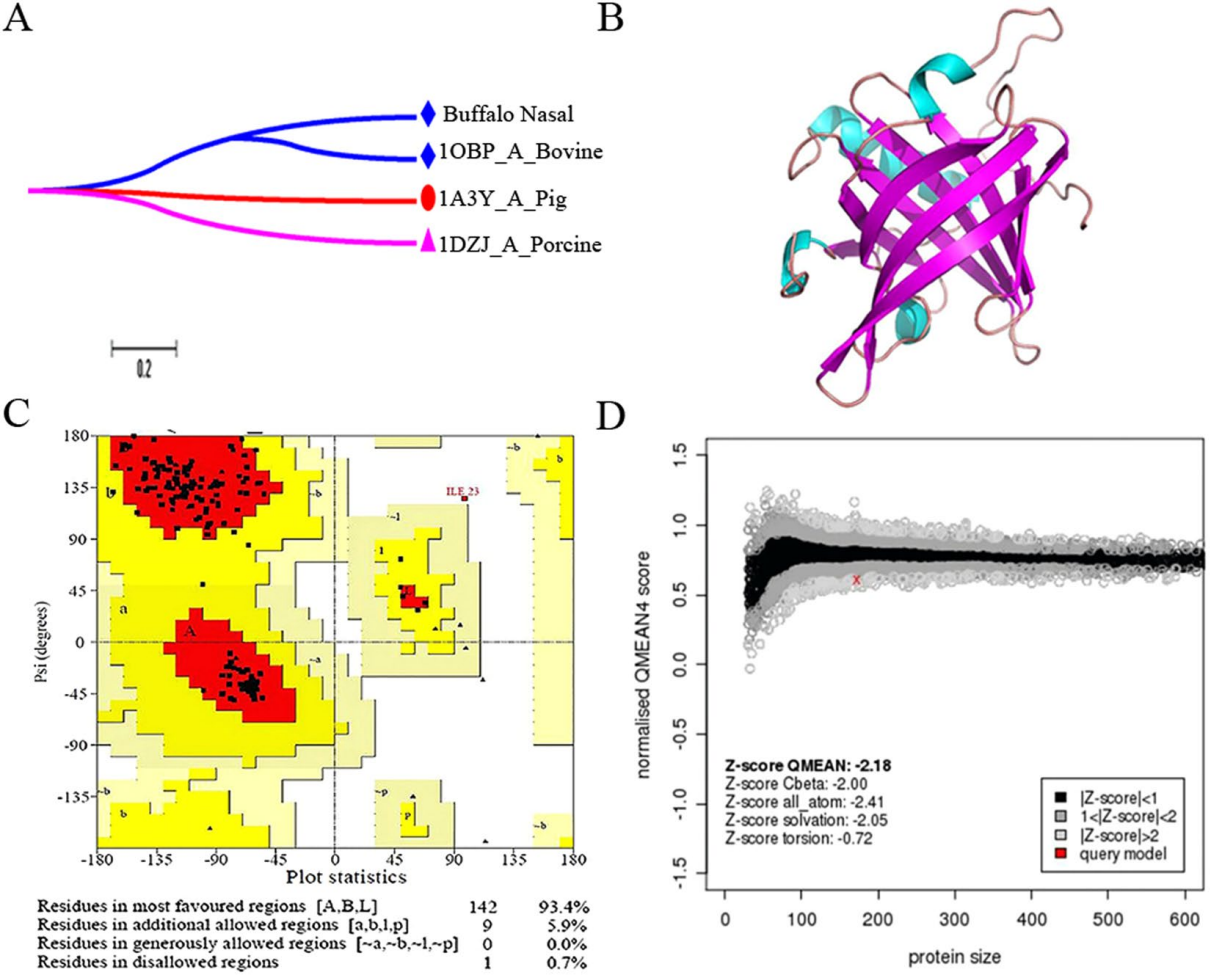
Molecular Modeling. (**A**) Phylogenetic tree of mammalian OBP including buffalo nasal OBP (bunOBP). Proteins are named using the PDB identity. Bovine and bunOBP are represented in same clad and show significant matching. (**B**) (PS)^2^-V2 was used to model the secondary structure of bunOBPand PyMol was used for visualization. The model shows continuous beta sheets with interconnecting loops/coils and looks like TIM barrel structure. (**C**) Ramachandranplot for bunOBP. It shows that 93.4% amino acid residues are located in most favored regions and 5.9% residues are located in the regions that are additionally allowed. (**D**) QMEAN score chart for the modeled bunOBP.

The un-rooted phylogeny for interspecies OBPs, viz., pig, porcine, bovine, and buffalo, were constructed. The maximum sequence resemblances of bovine OBP were observed with bunOBP and the clades were separated based on the bootstrap values. Pig and bovine OBP have the highest similarity and present as a neighbour members. Evolutionary diversity analysis confirmed that the bunOBP and bOBP appeared as co-clusters and so showed several similar conserved identities and motif regions (Figs 3D and 4A).

### bunOBP Modeling and Validation

The PDB model of chain A of 1BJ7 and 1OBP were taken as template for the structural modeling of bunOBP using MODELLER software (Version 9.8). Interestingly, the bunOBP revealed similar structural resemblances to templates. The bunOBP has continuous beta sheets with interconnecting loops/coils and its 3D structure depicts the TIM barrel shape, which is an outstanding feature of OBP^7^ (Fig. 4B).

The Ramachandran plot revealed that 93.4% residues occur in the most favored regions and 5.9% residues occur in additional allowed regions. Basically, a model with 90% of residues occurring in most favored regions is considered as the best (Fig. 4C). On the other hand, the model showed the least QMEAN score, −2.18 (Fig. 4D). Thirty binding sites were identified in bunOBP, in which the 30^th^ binding site revealed to be the largest and highly suitable for the ligands (Table S2). Hydrophobicity and pKa of bunOBP were analyzed and plotted with respect to each residue (Fig. S2).

### Superimposition of bunOBP

**Figure 5:**
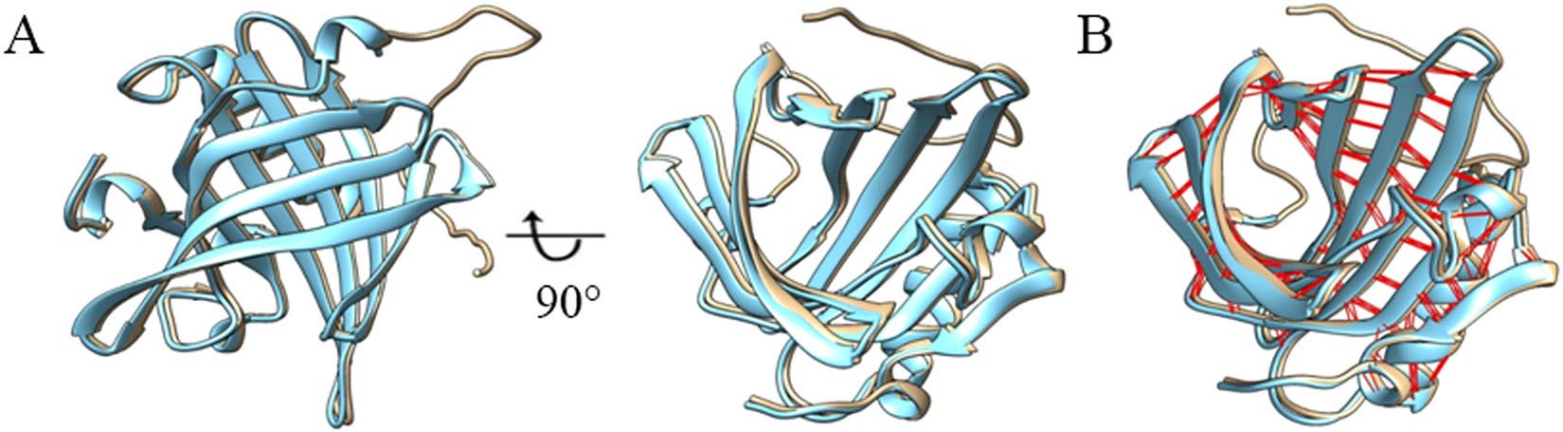
Structural Superimposition. (**A**) The cartoon-like ribbon representation of superimposed homology modeled bunOBP (grey) with the template (blue). Lateral and front view (90° rotations from bottom to top) of structures showing internal ligand binding cavity (**B**) The intra-model H-bond interactions are depicted in the superimposed bunOBP with red colour.

The structural model of bunOBP was superimposed with comparative template based on the sequence alignment with protein blocks (PBs) substitution matrix. The results revealed that the bunOBP model significantly matches with the template. The structural suitability of both the proteins showed root mean square deviation (RMSD) of 0.16, GDT_TS of 87.07. The structures and folding patterns of both proteins are similar. Interestingly, the bunOBP was superimposed fully with the template (Fig. 5A) and the inter-model H-bond interactions were depicted (Fig. 5B).

### bunOBP-Ligand Interaction Analysis

Physico-chemical properties of twenty chemical cues were screened and considered for visual inspection, in which top-docking scored compounds were listed along with their chemical formulae and molecular weights (g/mol) (Table 1). The bunOBP-chemical cues interaction was computed mainly in respect of absolute binding energy, H-bond-, van der Waals- and Pi-interactions (Table 2).

**Table 1:** Physico-chemical properties of putative chemical cues.

S. No	Compound Name	PubChem ID	Chemical formula	Molecular weight (g/mol)	H-bond donor	H-bond acceptor
1	1-Aminoanthracene	CID_11885	C14H11N	193.249	1	1
2	2-Isobutyl-3-Methoxypyrazine	CID_32594	C9H14N2O	166.224	0	3
3	Farnesol	CID_445070	C15H26O	222.372	1	1
4	1-octen-3-ol	CID_18827	C8H16O	128.215	1	1
5	1-iodo 2-methyl undecane	CID_545590	C12H25I	296.236	0	0
6	3,ethyl-2-methyl hexane	CID_86067	C9H20	128.259	0	0
7	p-Cresol	CID_2879	C7H8O	108.14	1	1
8	Oleic acid	CID_445639	C18H34O2	282.468	1	2
9	Pyridine	CID_1049	C5H5N	79.102	0	1
10	Undecanal	CID_8186	C11H22O	170.296	0	1
11	Diphenylmethanone	CID_3102	C13H10O	182.222	0	1

The compounds and the properties were collected from the PubChem server.

**Table 2:** The bunOBP-chemical cues interaction.

S. No	Compound Name	Glide Score (kcal/mol)	Total binding energy	H-bonds	Residues involved in Hydrogen bond	van der Waals interactions	Pi interactions
1	Oleic acid	−8.078	−103.251	2	Phe98, Glu99	Pro50, Leu51, Asn55, Ile73, Phe82, Gly84, Leu86, Ile96, Ile102, Leu104, Tyr118, Asn120, Val129	Cys53, Leu69, Phe71
2	p-Cresol	−8.029	−56.515	2	Asn55, Glu131	Thr34, Leu51, Phe71, Gly84, Leu104	Cys53, Leu69, Leu86, Ile96, Phe98, Ile102, Tyr118
3	1-Aminoanthracene	−7.392	−81.378	1	Asn55	Phe71, Phe82, Gly84, Leu86, Phe98, Asn120, Glu131	Leu51, Cys53, Leu69, Ile96, Ile102, Tyr118, Val129
4	Diphenylmethanone	−6.439	−70.707	—	—	Leu69, Phe71, Phe82, Gly84, Leu86, Phe98, Leu104, Asn120, Glu131	Cys53, Leu51, Ile96, Ile102, Tyr118
5	1-iodo 2-methyl undecane	−6.324	−67.785	—	—	Thr34, Cys53, Asn55, Ile58, Phe71, Leu86, Ile96, Phe98, Asn120, Glu131	Leu51, Leu69, Ile102, Phe116, Tyr118, Val129
6	3,ethyl-2-methyl hexane	−6.19	−58.023	—	—	Asn55, Leu104, Glu131	Leu51, Cys53, Leu69, Phe71, Leu86, Ile96, Phe98, Ile102, Tyr118, Val129
7	1-octen-3-ol	−5.706	−28.732	3	Asn55, Tyr118, Glu131	Leu69, Phe71, Leu86, Ile96, Phe98, Leu104, Asn120	Leu51, Cys53, Ile102
8	Farnesol	−5.534	−88.31	3	Asn55, Tyr118, Glu131	Lys44, Arg52, Cys53, Phe71, Leu86, Phe98, Asn120, Thr130, Ile164	Ala37, Ala39, Ile45, Leu51, Leu69, Ile96, Ile102, Leu104, Val129
9	Undecanal	−5.15	−58.602	2	Asn55, Glu131	Leu69, Phe71, Phe82, Leu86, Ile96, Phe98, Ile102, Leu104, Asn120, Val129	Leu51, Cys53, Tyr118
10	Pyridine	−4.321	−31.111	—	—	Leu51, Leu69, Phe71, Leu86, Leu104, Glu131	Cys53, Ile96, Phe98, Ile102, Tyr118
11	2-Isobutyl-3-Methoxypyrazine	−3.022	−65.811	1	Glu131	Asn55, Phe82, Gly84, Ile102, Val129	Leu51, Cys53, Leu69, Phe71, Leu86, Phe98, Leu104

The empirical values of molecular docking were retrieved by the best interactions based on Glide score, Total binding energy, and Hydrogen bond.

**Figure 6:**
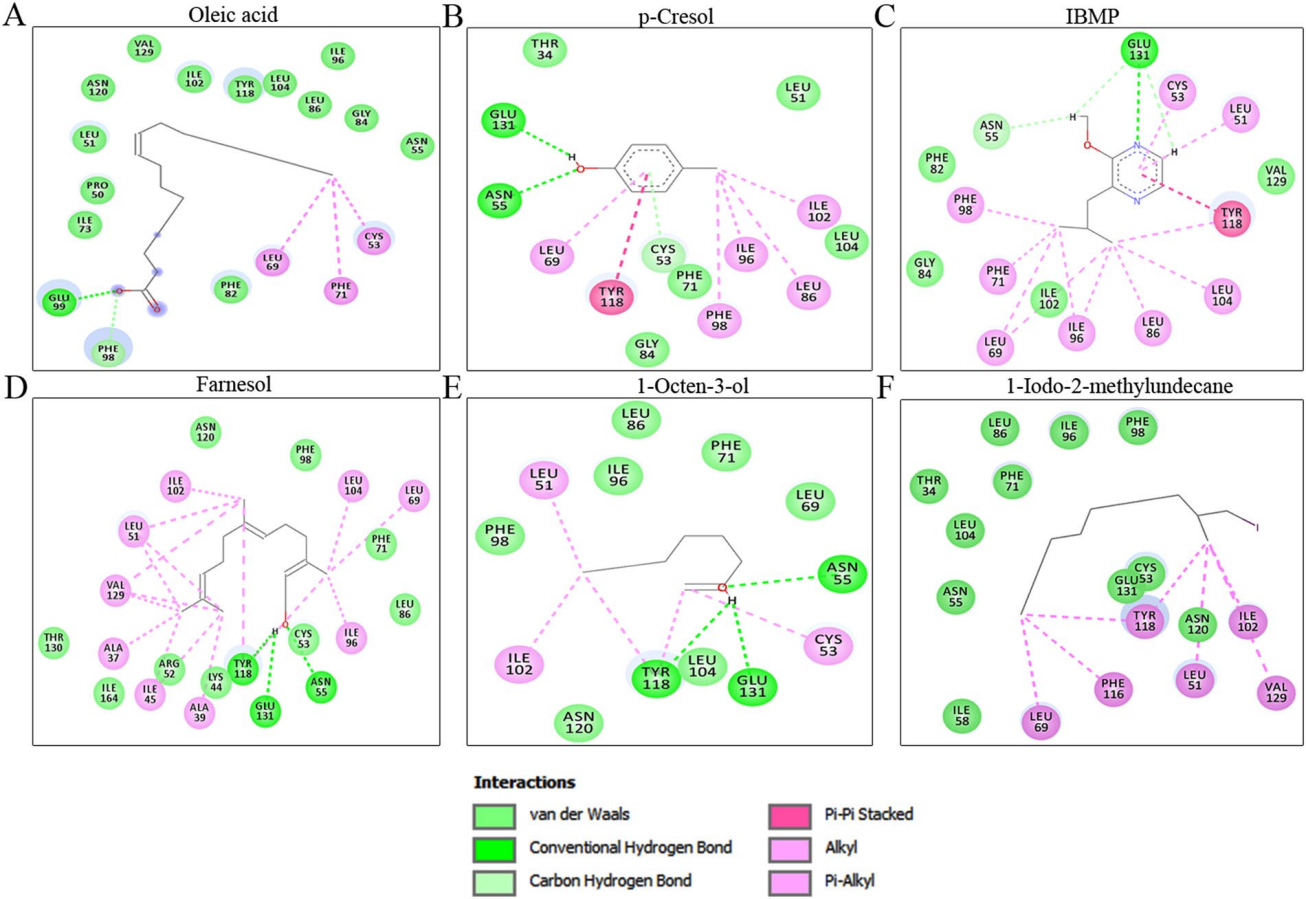
Molecular Docking. The chemical cues (**A**) Oleic acid, (**B**) p-Cresol, (**C**) 2-isobutyl-3-methoxypyrazine (IBMP), (**D**) Farnesol, (**E**) 1-Octane 3-ol, (**F**) 1-iodo-2-methlyundecane exhibit the highest binding interaction with bunOBP. The residues such as Asn55, Phe98, Glu99,Tyr118, and Glu131are best interacting residues with bunOBP.

Molecular docking analysis revealed oleic acid (CID_445639) and p-cresol (CID_2879) to have excellent binding affinity and energy toward bunOBP. Oleic acid showed the highest interaction, with glide score of −8.078 kcal/mol, and the binding energy was −103.251 kcal/mol, with two hydrogen bonds formed in Phe98 and Glu99 (Fig. 6A, Table 2). The next higher interaction was observed for p-cresol, which showed the second top glide score of −8.029 kcal/mol, with binding energy −56.515, and Asn55 and Glu131 participating in hydrogen bond interactions (Fig. 6B, Table 2). These two compounds were reported as estrus-specific, having commendable influence in male buffalo such as to provoke it to attempt to mate^25,26^.

The compound 2-isobutyl-3-methoxy pyrazine (IBMP) exhibited good binding interaction with hydrogen bond at Glu131(Fig. 6C, Table 2). Farnesol (CID_445070) showed glide score of −5.534 kcal/ mol with triple hydrogen bond interaction in Asn55,Tyr118 and Glu131 (Fig. 6D, Table 2). The compound 1-octen-3-ol bound with bunOBP with three hydrogen bonds at Asn55, Tyr118 and Glu131 (Fig. 6E, Table 2). Also, the ligand 1-Iodo2-methylundecane has van der Waals and alkyl interactions with bunOBP (Fig. 6F, Table 2). The docking of other listed ligands with bunOBP exhibited with good binding interaction (Fig. S3). Apart from hydrogen bond formation, the listed compound exhibited van der Waals- as well as Pi-interaction with the bunOBP (Table 2).

### Molecular Dynamics Simulations (MDS)

**Figure 7:**
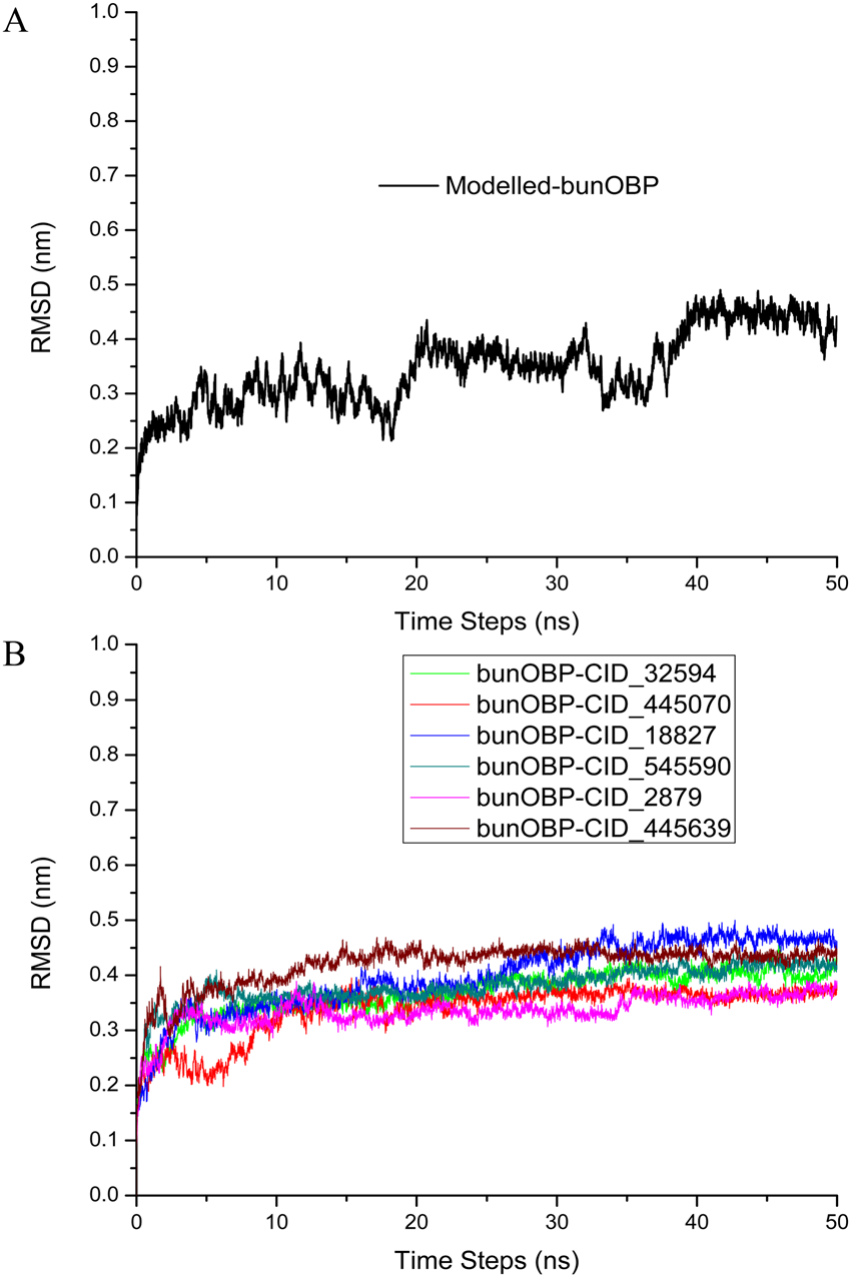
Molecular Dynamics Simulation. (**A**) RMSD of the backbone of comparatively modeled bunOBP shows a stable structure up to 50 ns. (**B**) RMSD of the backbone of protein-chemical cues complex exhibits a stable form without any significant conformational changes up to 50 ns.

In order to evaluate the stability of the modeled bunOBP structure and complexes, the top interacting six chemical cues have been studied using MD simulations for 50 ns for each complex. The modeled bunOBP backbone was found to have attained stability around 0.5 nm where the initial fluctuations were considered as the time taken for the equilibration and the RMSD (Root Mean Square Deviations) of the modeled bunOBP were worked out (Fig. 7A). We also evaluated the dynamic stability of all the complexes during the MD simulation, and the RMSDs of bunOBP backbone were analyzed and plotted.The results of MD simulation revealed that RMSD of the complexes almost reached equilibrium at ~3 ns. After the initial deviation, the complexes did not deviate further and showed consistent RMSD of around 0.5 nm throughout the simulation process. Further, the results showed that there was not much deviation throughout the simulation time for the top six compounds docked at the active site of bunOBP (Fig. 7B). Overall, the molecular dynamics of bunOBP revealed that the protein is highly stable in 50 ns simulations and ligand binding interactions are not affecting the structural conformation of bunOBP.

## Discussion

Odor perception and chemical communication are among the major life processes that help animals to identify conspecifics, same or opposite sex, and members of a different species and, therefore, constitute aspects of living together, colony formation, territory demarcation, mate selection, etc. The odorant/chemical cues are volatiles that are small molecules, produced by specific glands and released in minute quantities. They are transported/discharged in a form bound to specific proteins called pheromone-binding proteins. Also, when these odor molecules are perceived they invariably bind to specific OBPs and in this form they are presented to the specific receptors to elicit the appropriate response. Thus, the OBPs play a critical role in odor delivery and odor perception.

It is known that OBPs are secreted by different glands, and their roles depend upon the point from which discharged, say saliva, urine, nasal mucus, vaginal mucus, etc. For e.g., MUP, an urinary lipocalin in small mammals, is produced in the liver, transported in the blood as bound to the specific volatile, and sequestered into the urine to be discharged onto the external environment. However, proteins of homology similar to MUP have been identified in salivary glands and nasal septum as well. Thus, equivalent proteins are evolved in different body fluids that participate in pheromone shuttling such as (i) delivery and increasing the longevity of pheromone cues in urine, feces, vaginal secretion or saliva, and (ii) perception of pheromone cues through nasal secretions^27–29^. The OBP of nasal region, the subject of interest in this report, could have multiple functional roles in binding the odor molecule and presenting it to the specific olfactory receptor in the light of the barrier of nasal mucus^30^. Many reports have strongly established the presence of OBP in nasal region, which is concerned with glands and secretion^21,22,31^.

In view of the importance of OBP and its influence in odor perception, earlier we analysed the buffalo saliva when we conducted single dimensional gel electrophoresis and identified the presence of OBP encompassing its post-translational modifications^14^. On the other hand, nasal mucus is an important medium which has great functional significance in odor perception and chemosignal communication in the mammals^2,3^. Moreover, mating is stimulated by chemical cues, and a fair knowledge of how the buffalo finds its permissible mate can expound strategies to improve the reproduction management of the species. Having done that, now, we focus on OBPs, the soluble proteins, which would possibly act as carriers of pheromones to the olfactory receptors through the nasal mucus.

In our previous study pertaining to buffalo saliva, the electrophoretic separation showed low molecular weight protein as expressed prominently and it was interesting to note that the 21 kDa region stained prominently for glycoprotein and that particular region was matched with the OBP in mass spectrometry analysis^14^. Also, we have identified OBP at 21 kDa in a comparative salivary proteomic study during various phases of estrous cycle^32^. The present result substantiates that the high expression of low molecular weight proteins in nasal mucus of buffalo, especially the OBP, is matched at 21 kDa.

Understanding the functional significance of this nasal OBP in odor perception of buffalo would be rewarding. This is first time a nasal OBP has been discovered in a buffalo species adopting 2D proteome. Earlier, nasal OBPs have been purified and characterized in the cow^1^ rabbit, and pig^33^. The cow and rabbit OBPs lie around 19 kDa and at 4.7 pI but in the case of pig the protein was around 22 kDa and at 4.2 pI. Interestingly, the present two dimensional gel electrophoresis of buffalo nasal mucus revealed three spots at 21 kDa and at pI 4.2 which matched the OBP.

The protein spots at 21 kDa region were identified as OBP with several peptide matches. Further, that it is an OBP was emphasized with the support of *de novo* sequences, which was taken for the modeling of buffalo OBP. Before the modeling, the sequence of the protein was subjected to primary and secondary structure prediction using computational tools, which confirmed that the protein is the same as OBP in view of the similar molecular weight and pI. The physical and chemical properties are also the same as for OBP. The conserved residues of the OBP has been calculated and confirmed with the more conserved residual positions with other mammalian OBP sequences in MSA, this protein depicts higher conservation with bovine OBP. The characteristic feature of the OBP such as -GXW-^3,34,35^ motif and conserved residues are present in the bunOBP. The phylogenetic analysis also demonstrated that the bunOBP has the highest similarity with bovine OBP. The porcine OBP was found as an adjacent clad towards OBP members.

The first 3D structure of bovine OBP was demonstrated by Tegoni *et al*.^22^. A monomeric structure of porcine OBP (pOBP) has also been reported^36^. However, the basic structural information in respect of buffalo nasal OBP is not fully clear. Hence, based on the present data, the comparative homology model was developed for buffalo nasal OBP(bunOBP). Apparently, the model showed the favorable feature of OBP and ligand binding nature. Interestingly, the most important structure, beta (TIM) barrel^7^, is depicted in bunOBP, which would provide a favourable binding position to the odor/chemical cues. The model thus developed showed 30 binding sites, which are capable of facilitating accommodation of odorants. Furthermore, the bunOBP model has been validated and the model has 93.4% amino acid residues positioned around the most favored regions.

For confirming the binding interaction with the chemical cues, the experiment was conducted to find the binding with the different ligands already known. 2-Isobutyl-3-methoxypyrazine, a green-type odor molecule, analyzed in this study, also has been found to have good interaction with OBPs in general and, hence, an association of this ligand with any new OBP was evaluated. Moreover, farnesol has been reported as a good repellent in aphids and the OBP3 and OBP7 are the proteins responsible for mediating the perception of the alarm pheromone^37^. Territorial marking, mating, and individual recognition are the important pheromone effects of farnesol in elephants and various insects^38^. It is interesting to note that α and β farnesenes, in male mouse urine, have been identified as pheromones for communication among the individuals^39^.1-iodo-2 methyl undecane has been identified in mice urine specifically during the estrus period and further confirmed as estrogen-dependent, which also enhances the reproductive activities of male mice^40^. The present computational analysis provides strong evidence for these molecules to bind and interact with bunOBP.

Especially, the pheromone compounds, i.e., p-cresol (4-methyl phenol) and oleic acid (9-octadecenoic acid) in urine^26^, and p-cresol alone in feces^25^ have been reported as estrus-specific in buffalo. In particular, both the compounds are consistently present in more than one body exudates and are proved to be pheromone compounds released in estrus buffalo. The reports available authenticate these estrus-specific volatiles to act as attractants and inducers of mounting behavior in buffalo bull^26,41^. Combinations of these two pheromones are known to elicit bull sexual behavior^42^. The present molecular-docking and -dynamics simulations results expound that the compounds oleic acid and p-cresol are best posed to interact and bind with bunOBP. Thus, herein we affirm the presence of an estrus-specific compound in the body exudates of estrus she-buffalo which could be perceived by the he-buffalo with the help of bunOBP as a shuttle, and the combined molecule may lead to signal transduction in the olfactory system.

In conclusion, this is the first report of experimental evidence for the presence of an odorant binding protein in the nasal mucus of buffalo. Further, the computational study substantiates that this protein would possibly engage in odor perception and sexual communication in buffalo.

## Materials and Methods

### Sample Collection and Preparation

The animals were maintained in the farm at Tamil Nadu Veterinary College and Research Institute (TANUVAS), Namakkal, India. Nasal mucus was collected from Murrah buffaloes, *Bubalus bubalis* (n = 6), that were healthy, using finger protected by sterile gloves as a swab (Kimberly-Clark, Rosewell, USA). The mucus adsorbed on the glove was collected in sterile tubes. Then, the mucus was homogenized using a Borosil glass homogenizer and centrifuged at 12,000 × g for 15 min at 4 °C. The supernatant was collected and stored at −80 °C until further analysis. The Institutional Animal Ethics Committee of TANUVAS had approved the animal handling and procedure for sample collection. The methods were carried out in accordance with the relevant guidelines.

### TCA-Acetone Precipitation

The supernatant as above was treated at −20 °C for 1 h with two volumes of 10% TCA (Trichloroacetic acid) (w/v) prepared in acetone containing 20 mM DTT (Dithiothreitol). The mixture was subjected to centrifugation at 12,000 × g for 20 min at 4 °C.The pellet was washed three times with ice-cold acetone, air-dried, re-suspended in buffer containing 10mMTris-HCl (pH 8.0), 1 mM EDTA and 1% SDS, and boiled for 5 min at 95 °C^43^. Bradford^44^ method was adopted to determine the concentration of protein. The protein thus separated was stored at −80 °C until use.

### SDS-PAGE

Protein analysis was performed adopting SDS-polyacrylamide gel electrophoresis (SDS-PAGE) for which 12.5% resolving gel (12 × 14 cm) and 4% stacking gel were used. The concentrated protein samples were diluted in 1x loading buffer [0.08 M Tris-HCl (pH 6.8), 2.7% SDS, 13.7% glycerol, 0.97 M β-mercaptoethanol, 0.3% bromophenol blue] and boiled at 100 °C for 3 min prior to loading onto the gel^45^. Tris-glycine-SDS buffer containing 0.3% Tris, 1.44% glycine and 0.1% SDS formed the running buffer. Electrophoresis was performed at 10 mA current for 6–7 h until the tracking dye reached the bottom of the resolving gel. The gel was visualized after glutaraldehyde-silver staining.

### Isoelectric Focusing

The precipitate of the mucus proteins was mixed with an equal volume of UTC buffer containing 6 M urea, 3 M thiourea, 8% CHAPS, 50 mM DTT, 2% IPG buffer (GE Healthcare), and 0.004% bromophenol blue and incubated for 30 min in ice. The sample was diluted to the required volume using UTC rehydration buffer (UTCRB) [7 M urea, 2 M thio-urea, 4% CHAPS, 0.5% ampholytes, 50 mM DTT, 1% IPG buffer, and 0.004% bromophenol blue]. After loading of samples, the IPG strips were focused in EttanIPGphor 3IEF after 16 h of passive rehydration. The program used for focusing 11 cm IPG strips was 0 V-1 h; 30 V-11 h; 200 V-1 h; 1000 V-1 h (Grad); 5000 V-2 h (Grad); 8000 V-1 h (Grad); 8000 V-7 h. The strips were stored at −80 °C until the second dimension electrophoresis was conducted.

### Second Dimension Electrophoresis

In order to reduce and alkylate the proteins, the strips were subjected to two step equilibration (15 min each) with DTT and IAA (2-Iodoacetamide), respectively, at room temperature. The strips were then placed on top of a 12.5% polyacrylamide gel slab (14 cm × 14 cm × 1 mm) and sealed using 0.5% agarose prepared in 1x electrophoresis buffer. The upper tank buffer was Tris-glycine-SDS containing 0.6% Tris, 2.88% glycine and 0.2% SDS; the lower tank buffer was Tris-glycine-SDS buffer containing 0.3% Tris, 1.44% glycine and 0.1% SDS^42–45^. The electrophoresis condition for a single gel was 0.5 W for 45 min and 2 W for 5–6 h until the tracking dye reached the bottom of the gel.

### Staining Methods

The gels were fixed in a mixture containing methanol, acetic acid and water in 4:1:5, for 1 h to overnight, and stained with freshly prepared colloidal Coomassie brilliant blue G-250, for 6 h to overnight^46^. The stained gel was washed until the background stain was completely cleared.

### Trypsin In-Gel Digestion

Protein spots of interest were cut off from the gel and the dye was selectively removed by repeated incubation in 100 µL of 25 mM NH_4_HCO_3_/50% (v/v) acetonitrile (1:1) at 56 °C for 30 min. A Speed-Vac (Thermo Fisher Scientific,Waltham, MA, USA) was used to dry the gel spot. Further alkylation and reduction were carried out as per the protocol of Muthukumar *et al*.^32^. The gel spots were incubated at 37 °C in 25 mM NH_4_HCO_3_ containing100 ng modified trypsin (Promega, Mannheim, Germany) for overnight. The peptide digests were separated and dried in Speed-Vac. The dry peptides were subjected to mass spectrometric analysis after re-suspension in 0.1% formic acid.

### Mass Spectrometric Analysis

LTQ-Orbitrap (Discovery) hybrid mass spectrometer LC-MS/MS (ThermoElectron Corporation, San Jose, CA, USA) which couples with a nano-HPLC (Agilent Technologies 1200 Series,Waldbronn, Germany) was used. The Agilent C18 column (100 × 0.075 mm; 3.5 µm particle diameter) was used for the fractionation. Formic acid in water (0.1%) and formic acid in acetonitrile (0.1%) were the two mobile phases used in the experiment. The flow rate of the pump was 0.5 µL/min. The MS spectrum (Survey Scan) was acquired over the acquisition range m/z 200–2000 at high resolution (M/∆M, 60,000 full width half maximum). Precursor ions were selected for the MS/MS scan. Further, the MS/MS spectrum was obtained for the fragment ions generated by collision-induced dissociation^32,47^.

### Sequence Analysis in Database

The bunOBP sequence was obtained from Mascot, and sequence matches were identified using Basic Local Alignment Search Tool (BLAST) from UniProt database (http://www.uniprot.org/). The highly matched protein ID was Q0IIA2_ BOVINE, similar to odorant-binding protein.

### Physicochemical Properties and Amino Acid Analysis

The amino acid (AA) composition of bunOBP and the various physico-chemical properties were derived from the primary protein sequence and computed adopting ProtParam tool accessible from EXPASY bioinformatics resource portal (http://web.expasy.org/protparam/). The tool was used to confirm molecular weight, theoretical pI, atomic composition, extinction coefficient, estimated half-life, instability index, aliphatic index and grand average of hydropathicity (GRAVY)^48^.

### Secondary Structure and Conserved Map

SOPMA (Self-optimized prediction method with multiple alignments) tool was used to secondary structure prediction of protein including alpha helix, extended strand, beta turns, beta bridge and random coils of bunOBP (http://npsa-pbil.ibcp.fr/cgi-bin/npsa_automat.pl?page=npsa_sopma.html)^49^. Conserved residues were analysed, and the residue conservation was mapped for bunOBP using ConSurf server (http://consurf.tau.ac.il/verify.php)^50^.

### MSA and Phylogeny

The OBP sequence of bovine, porcine and pig were selected and retrieved from non-redundant database and PDB (protein data bank) website. The bunOBP sequence was aligned with the collected sequences for identification of identical residues and conserved motifs through ClustalW2 (http://www.ebi.ac.uk/Tools/msa/clustalw2/), available in EMBL-EBI webservices^51^. Extra indels in the sequence alignment were manually edited adopting MEGA 5.0^52^ and visualized using ESPript server (http://espript.ibcp.fr/ESPript/ESPript/). The phylogentic tree was constructed for mammalian OBP to identify neighboring members of bunOBP. The generated alignment was followed in the prediction of phylogenetic tree through neighbor joining (NJ) method and it was viewed using MEGA 5.0.

### Template Selection

The bunOBP sequence was subjected to BLASTp against protein sequences from PDB (http://www.rcsb.org) and the maximum matched sequences were identified based on e-value thresholds. The first hit was showed the best template for the construction of the structural model. SWISS-MODEL, an open source fully automated web-based server, was adopted to annotate the bunOBP sequence alignment using three dimensional (3D) structural information of template^53^. The template identification was performed adopting SWISS-MODEL workspace, and the first hit exhibited good sequence matches of structural information in template selection.

### Homology Modeling

The X-ray crystallographic structure of bovine lipocalins was used as the template for construction of homology model. Finally, the alignments were generated between of query (bunOBP), and template structure and the alignment was used to construct the model made by MODELLER software (Version 9.8)^54^. A set of 20 models were generated and retrieved from MODELLER. Among the 20 models, the top five low energy optimized models were selected and subjected to assessment of the stereo-chemical quality of modeled structure by analyzing residue-by-residue geometry with validation using phi/psi value acquired by Ramachandran plot analysis from PROCHECK, accessible in SAVeS server (https://www.ebi.ac.uk/thornton-srv/software/PROCHECK/)^55^. PROCHECK tool was used to calculate the allowed and disallowed regions and also, to determine the helices, strands, coils in the structural model. The model that attained the maximum number of residues in the allowed and additional allowed regions of Ramachandran plot was considered as the best one for molecular docking, and additional validation was executed by QMEAN Server for model quality estimation (http://swissmodel.expasy.org/qmean/cgi/index.cgi)^56^. The final model was energy-minimized using SWISS-PDB viewer to obtain stable structure^57^. Further, molecular visualization of the constructed model was performed using PyMol software (The PyMOL Molecular Graphics System, Version 0.99rc6, Schrödinger) and UCSF Chimera (http://www.cgl.ucsf.edu/chimera/). Q-SiteFinder server was used to predict the possible binding sites of modeled protein (http://www.modelling.leeds.ac.uk/qsitefinder/)^58^.

### Structural Superimposition of bunOBP

The homology modeled bunOBP was superimposed with the corresponding template for prediction of folding pattern and comparison of structures based on similarity and backbone conformation. The superimposition was performed by iPBA, a webserver (http://www.dsimb.inserm.fr/dsimb_tools/ipba/). The proteins were superimposed by structural alignment and dynamic programming of PBs substitution matrix.

### Formulation and Optimization of Ligands

The available mammalian chemical cues were analysed from PDB and research articles adopting data mining approach. Thus, the ligands were downloaded as 3D structures (.sdf file format) and retrieved from PubChem database (http://pubchem.ncbi.nlm.nih.gov/)^59^. Further, the ligands were submitted for energy minimization using ACD/ChemSketch (Version 10.0) (Advanced Chemistry Development, Toronto, ON, Canada, www.acdlabs.com).

### Molecular Docking

The molecular docking simulation was done using one of the most suitable methods for performing receptor-ligands docking, the commercialized GLIDE software package (Grid-based Ligand Docking with Energetics-Schrödinger- *Maestro, version 9.3, Schrödinge*r*, LLC, New York, NY, 2012)*^60,61^. It is easy to run the docking simulation through the graphical user interface in GLIDE (Schrödinger suite). The bunOBP was adopted for analysis of receptor-ligand interaction by choosing the best fit ligand. The receptor (bunOBP) grid was created by the receptor generation tool in Glide application. The ligands were then docked to the modeled protein using Glide suite. The best fit ligands were selected based on virtual screening with receptor for docking. Finally, the docked conformers were assessed using GLIDE score and the best poses were generated on output. The residual interactions within the binding site are proportional to the odorant size; it is not related to the binding affinity with protein^62^. The additional computational procedures for docking were followed according to Golebiowski *et al*.^62^ to support the protein-ligand interaction and structural conformation stability.

### Molecular Dynamics Simulations

Protein and ligand structures used in GROMACS 5.1.4 package simulation must be complete for all-atom 3D structures with a reasonable geometry. The topology files for the protein were generated using the automated topology builder in the framework of GROMOS96 53a6 force field for protein-ligand complex^63,64^. The ligand topologies were generated by the PRODRG server^65^. The complexes were immersed in a cubic box containing simple point charge (SPC) water molecules, and appropriate counter ions, Na^+^ and Cl^−^, were added in order to neutralize the net charge of the system^66^. The long-range electrostatic interactions were calculated with the Particle Mesh Ewald (PME) method^67,68^. Bond lengths involving hydrogen atoms were constrained by using the Linear Constraint Solver (LINCS) algorithm^69^. Further, NVT was performed for 100 ps to equilibrate the system with protein and ligand for constant volume, pressure (1 atm) and temperature (300 K). The final MD run was set to 50 ns for each ligands complex with modeled bunOBP, and trajectories were saved for further analysis using GROMACS analysis tools.

## Electronic supplementary material

Figure S1 S2 S3 Table S1 S2
