# Author Correction: Buffalo nasal odorant-binding protein (bunOBP) and its structural evaluation with putative pheromones

**DOI:** 10.1038/s41598-018-29894-6

**Published:** 2018-08-01

**Authors:** Subramanian Muthukumar, Durairaj Rajesh, Ramu Muthu Selvam, Ganesan Saibaba, Suvaiyarasan Suvaithenamudhan, Mohammad Abdulkader Akbarsha, Parasuraman Padmanabhan, Balazs Gulyas, Govindaraju Archunan

**Affiliations:** 1 Center for Pheromone Technology (CPT), Department of Animal Science, Bharathidasan University, Tiruchirappalli, 620024 Tamil Nadu India; 2 Center for Animal Research, Training and Services (CAReTS), Central Inter-Disciplinary Research Facility (CIDRF), Mahatma Gandhi Medical College & Research Institute campus, Pillaiyarkuppam, Puducherry 607402 India; 3 Research Institute in Semiochemistry and Applied Ethology (IRSEA), Quartier Salignan, 84400 APT France; 4 Present Address: Winro Research Institute of Biological Sciences, winro Science Research Foundation, Tiruchirapalli, 620007 Tamil Nadu India; 5 Department of Bioinformatics, Bharathidasan University, Tiruchirappalli, 620 024 Tamil Nadu India; 6 Mahatma Gandhi-Doerenkamp Centre, and Department of Animal Science, Bharathidasan University, Tiruchirappalli, 620024 Tamil Nadu India; 7 Present Address: National College (Autonomous), Tiruchirappalli, 620001 Tamil Nadu India; 8 Lee Kong Chian School of Medicine, Nanyang Technological University, Singapore, 636921 Singapore

Correction to: *Scientific Reports* 10.1038/s41598-018-27550-7, published online 19 June 2018

The original version of this Article contained an error in Affiliation 8, which was incorrectly given as ‘The Lee Kong Chian School of Medicine, Nanyang Technological University, Singapore 636921, Singapore’. The correct affiliation is listed below:

‘Lee Kong Chian School of Medicine, Nanyang Technological University, Singapore 636921, Singapore’

In addition, in the Supplementary file ‘Figure S1 S2 S3 Table S1 S2’, the Present Addresses for the authors Ramu Muthu Selvam and Mohammad Abdulkader Akbarsha were omitted.

These errors have now been corrected in the HTML and PDF versions of the Article, and in the accompanying Supplementary Information file.

