# Supplementary material for: Buffalo nasal odorant-binding protein (bunOBP) and its structural evaluation with putative pheromones
Source: Sci Rep. 2018 Jun 19;8:9323. doi: 10.1038/s41598-018-27550-7 (PMC6008301; doi:10.1038/s41598-018-27550-7)
Supplement: Supplementary file 1 — Figure S1 S2 S3 Table S1 S2 [file 41598_2018_27550_MOESM1_ESM.doc]

**Buffalo nasal odorant-binding protein (bunOBP) and its structural evaluation with putative pheromones**

**Subramanian Muthukumar^1, 2^, Durairaj Rajesh^1, 3^, Ramu Muthu Selvam**^1,4^**, Ganesan Saibaba^1^, Suvaiyarasan Suvaithenamudhan^5^, Mohammad Abdulkader Akbarsha^6,7^, Parasuraman Padmanabhan^8*^, Balazs Gulyas^8^, Govindaraju Archunan^1*^**

^1^Center for Pheromone Technology (CPT), Department of Animal Science, Bharathidasan University, Tiruchirappalli, 620024, Tamil Nadu, India.

^2^Center for Animal Research, Training and Services (CAReTS), Central Inter-Disciplinary Research Facility (CIDRF), Mahatma Gandhi Medical College & Research Institute campus, Pillaiyarkuppam, Puducherry, 607402, India.

^3^Research Institute in Semiochemistry and Applied Ethology (IRSEA), Quartier Salignan, 84400, APT, France.

^4^ Present address: Winro Research Institute of Biological Sciences, winro Science Research Foundation, Tiruchirapalli, 620007, Tamil Nadu, India.

^5^Department of Bioinformatics, Bharathidasan University, Tiruchirappalli, 620024, Tamil Nadu, India.

^6^Mahatma Gandhi-Doerenkamp Centre, and Department of Animal Science, Bharathidasan University, Tiruchirappalli, 620024, Tamil Nadu, India

^7^Present address: National College (Autonomous), Tiruchirappalli, 620001, Tamil Nadu

^8^Lee Kong Chian School of Medicine, Nanyang Technological University, Singapore 636921. Singapore.

***Correspondence:**

1. Dr. G. Archunan, UGC-BSR Faculty fellow, Center for Pheromone Technology, Department of Animal Science, Bharathidasan University, Tiruchirappalli– 620024, Tamil Nadu, India.

Phone: 91-431-2407040, Fax: 91-431-2407045, Email: [archunan@bdu.ac.in](mailto:archunan@bdu.ac.in)

2. Dr. P. Padmanabhan, Deputy Director, Lee Kong Chian School of Medicine, Nanyang Technological University, 59 Nanyang Drive, Singapore 636921

Phone: +65-64789058, Email: [ppadmanabhan@ntu.edu.sg](mailto:ppadmanabhan@ntu.edu.sg)

**Supplementary Figures**

**Figure S1**. **Structural conservation of bnOBP using FirstGlance in Jmol**. (A) Visualize the conservation pattern in bnOBP with existing OBP models (B) The highest conserved residues positions were depicted in α-helices, β-sheets, and random coils based on conservation scale.

B


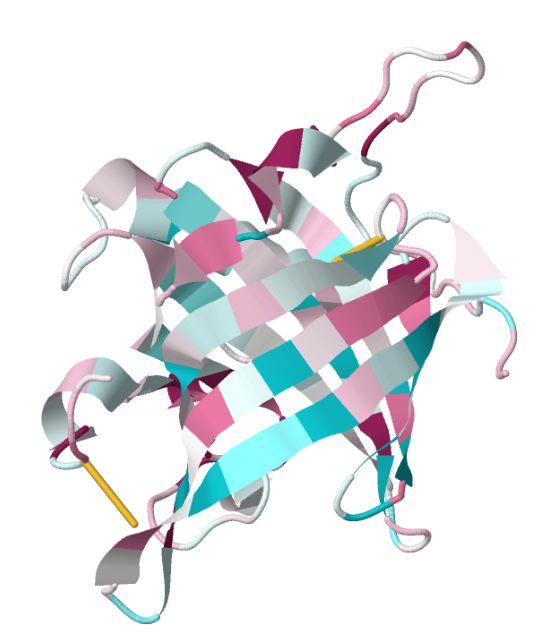

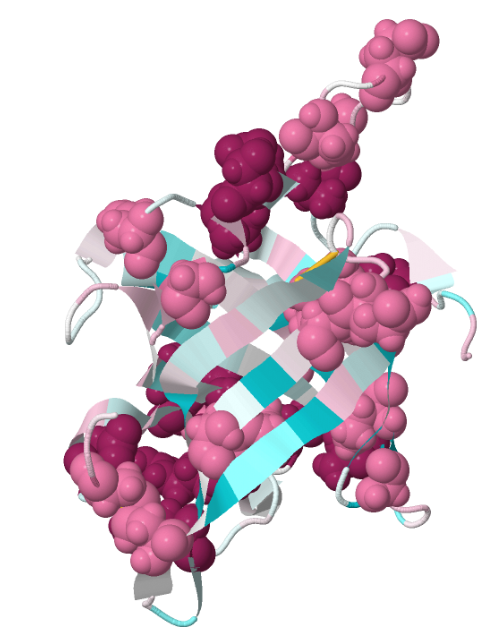


A


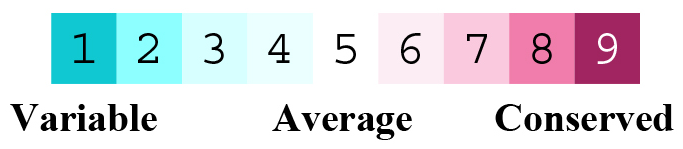


**Figure S2**. **Heatmap and hydrophobicity plot.** A) The heatmap illustration of homology modeled bnOBP showed the structural property such as hydrophobicity, pKa by rank based colour scale. B) The plot showed the hydrophobic residual fluctuations of bnOBP, which was predicted using HPhob. /Kyte & Doolittle algorithm in ProtScale server.


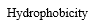

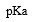

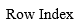

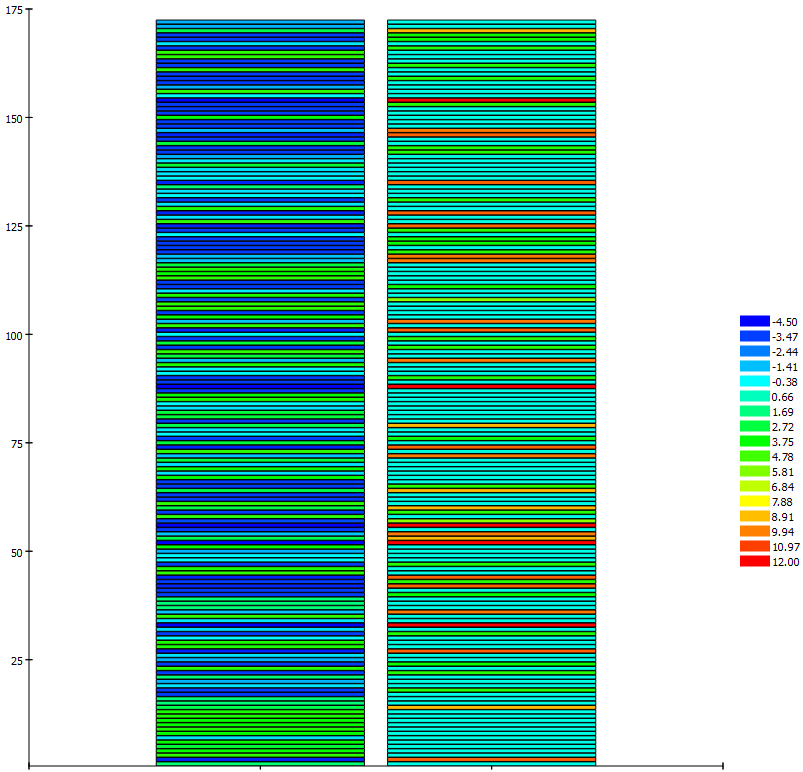

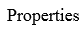


A

B


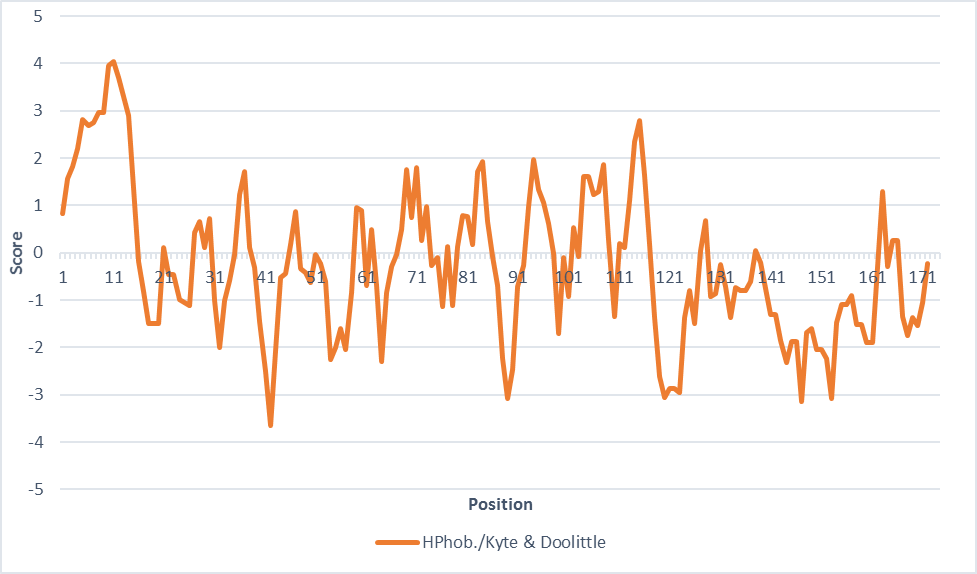


**Figure S3**. **Molecular docking.** The figure shows the 2D expression of interaction between ligands and bnOBP. A. 1-Aminoanthracene, B. Diphenylmethanone, C. 3, ethyl-2 methyl hexane, D. Undecanal and E. Pyridine.

**
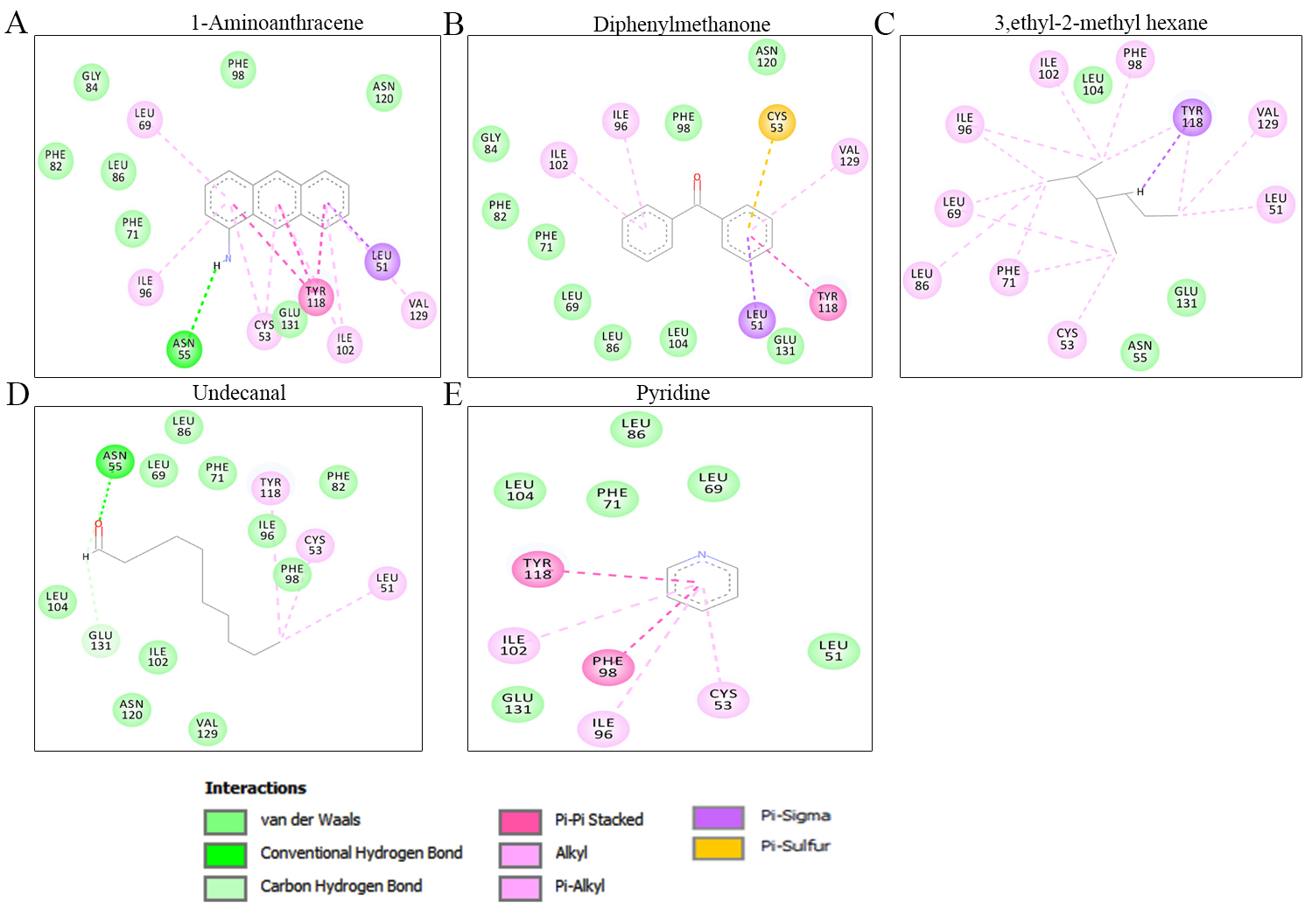
**

**Supplementary Tables**

**Tables S1. bnOBP sequence information.**

| **Amino Acid** | **Number of Residues** | **Percentage of residues** |
| --- | --- | --- |
| Ala_(A) | 7 | 4.10 |
| Arg_(R) | 5 | 2.90 |
| Asn_(N) | 11 | 6.40 |
| Asp_(D) | 8 | 4.70 |
| Cys_(C) | 6 | 3.50 |
| Gln_(Q) | 9 | 5.20 |
| Glu_(E) | 19 | 11.00 |
| Gly_(G) | 12 | 7.00 |
| His_(H) | 2 | 1.20 |
| Ile_(I) | 17 | 9.90 |
| Leu_(L) | 12 | 7.00 |
| Lys_(K) | 10 | 5.80 |
| Met_(M) | 1 | 0.60 |
| Phe_(F) | 10 | 5.80 |
| Pro_(P) | 7 | 4.10 |
| Ser_(S) | 7 | 4.10 |
| Thr_(T) | 9 | 5.20 |
| Trp_(W) | 1 | 0.60 |
| Tyr_(Y) | 8 | 4.70 |
| Val_(V) | 11 | 6.40 |
| Pyl_(O) | 0 | 0.00 |
| Sec_(U) | 0 | 0.00 |

The sequence information was retrieved from the PROTPARAM tool in EXPASY bioinformatics resource portal.

**Table S**2. Shows the binding site area and volume of bnOBP.

| **ID** | **Area** | **Vol** | **Amino acid residues with position** |
| --- | --- | --- | --- |
| **30** | 982.5 | 1731.3 | LYS2,ILE3,LEU4,PHE5,LEU6,SER7,VAL9,VAL12,VAL13,CYS14,ALA15,THR19,ALA21,GLU22,ILE23,PRO25,VAL28,ASN63,CYS64,LEU67,ARG88,GLU90,GLY91,GLY92,VAL93,TYR94,TYR103,GLN105,ILE106,ILE107,HIS108,TYR117,GLU119,ILE126,LYS128,GLN149,LEU150,GLU153,ARG154 |
| **29** | 344.3 | 344 | ALA37,ILE45,LEU51,CYS53,ASN55,LEU69,PHE71,PHE82,LEU86,ILE96,PHE98,ILE102,LEU104,TYR118,ASN120,VAL129,GLU131 |
| **28** | 94.8 | 84.2 | LYS2,LYS101,ILE102,TYR103,GLU119,ASN120,ASP121,ILE126 |
| **27** | 116.6 | 114.7 | GLU47,GLY48,GLY49,PRO50,ARG52,ILE73,LYS74,PHE75 |
| **26** | 76.5 | 62 | ARG33,THR34,TYR54,ASN55,ARG56,ILE164,ILE165,ASP168 |
| **25** | 91.7 | 62.6 | HIS108,ILE113,VAL115,GLU143,LYS146,TYR147 |
| **24** | 32.5 | 30.4 | LYS74,GLY77,THR78,CYS79,ASP169 |
| **23** | 78.3 | 47.1 | ILE113,VAL115,GLY132,PHE139,GLU143,TYR147 |
| **22** | 63.3 | 39.6 | ILE107,TYR117,THR130,LEU150,ASN151,ARG154,ILE156 |
| **21** | 36.2 | 20.1 | VAL28,ILE106,VAL109,LEU114 |
| **20** | 88.1 | 57.6 | TRP32,THR34,ASN55,ILE58,PHE116,GLU131,SER133 |
| **19** | 45.1 | 29.3 | HIS108,THR110,ASN112,ILE113,GLU143,LYS146 |
| **18** | 30.3 | 18.2 | GLU22,ILE23,HIS108,VAL109 |
| **17** | 24 | 15.5 | MET1,GLU65,GLN66,GLN87,ARG88 |
| **16** | 32.2 | 16.9 | ALA37,LEU51,ARG52,CYS53,GLU131,ILE164 |
| **15** | 16.2 | 10.7 | MET1,LYS2,PHE95,LYS101 |
| **14** | 39.7 | 18.6 | GLU142,GLU143,LYS146 |
| **13** | 25 | 18 | ILE35,TYR36,THR137,SER138,ASN163,ILE165 |
| **12** | 9.7 | 7.7 | LEU8,VAL9,VAL12 |
| **11** | 18.3 | 11.5 | ILE73,GLN80,PHE82,PHE98 |
| **10** | 28.8 | 14.4 | LYS27,THR110,ASP111,LYS135 |
| **9** | 37.7 | 19.2 | PRO25,ASN63,CYS64,ARG88 |
| **8** | 29.8 | 15.2 | LYS2,GLN89,PHE95,TYR103 |
| **7** | 27.9 | 13.7 | PHE82,ILE96,GLU97,PHE98 |
| **6** | 29.2 | 14.2 | ALA39,ILE45,LEU51,VAL129 |
| **5** | 22.3 | 11.7 | ASN41,GLU43,LYS44,LYS125 |
| **4** | 26.3 | 12.6 | TYR117,VAL129,ILE156 |
| **3** | 34.1 | 17.9 | LEU69,PHE71,GLY84,ILE96 |
| **2** | 15.9 | 7.9 | GLN145,LYS146,GLN149 |
| **1** | 15.9 | 8.1 | ASP40,LYS128,GLY155 |

Foot note: 30^th^ binding site consumes largest binding volume compared to all other and which accommodate the suitable ligands in the internal cavity.
